# The hypertriglyceridemic waist phenotype is associated with fatty liver and glycometabolic profiles in overweight and obese adults: a cross-sectional study

**DOI:** 10.1038/s41598-021-00825-2

**Published:** 2022-02-14

**Authors:** Meiling Zhou, Feifei Li, Haokai Tang, Si Wu, Li Meng, Yanhui Dong, Fei Wang, Binh Quach, Yide Yang, Jun Ma, Julien Steven Baker

**Affiliations:** 1 Key Laboratory of Molecular Epidemiology of Hunan Province, School of Medicine, Hunan Normal University, Changsha, China; 2 Department of Sport, Physical Education and Health, Centre for Health and Exercise Science Research, Hong Kong Baptist University, Hong Kong, China; 3 Institute of Child and Adolescent Health, School of Public Health, Peking University Health Science Center, Beijing, China; 4 Hunan Provincial Center for Disease Control and Prevention, Changsha, Hunan China

**Keywords:** Metabolic syndrome, Obesity, Gastroenterology, Hepatology

## Abstract

The present study aimed to distinguish different hypertriglyceridemic waist phenotypes and relevant risks of developing fatty liver and abnormal glycometabolic profiles in overweight/obese adults. A total of 1221 Chinese adults with mean (standard deviation [SD]) age of 37 (9) years, 37.3% males and 62.7% females, body mass index (BMI) of 29.0 (4.0) kg/m^2^, triglyceride (TG) 2.04 (1.45) mmol/L, and waist circumference (WC) 95.8 (10.7) cm were included and classified into four phenotypes: normal TG & normal WC (N-N); normal TG & high WC (N-WC); high TG & normal WC (TG-N); high TG & high WC (TG-WC). Participants in TG-WC group had the highest BMI, WC, blood pressure (BP), insulin, Homeostatic Model Assessment of Insulin Resistance (HOMA-IR), glycosylated hemoglobin (HbA1c), low-density lipoprotein cholesterol (LDL), and fatty liver. Participants within N-WC group had a significantly higher risk of fatty liver (adjusted OR 3.50 [95% CI 2.05–5.97]), as well as TG-N (adjusted OR 2.59 [95% CI 1.61–4.16]) and TG-WC (adjusted OR 4.12 [95% CI 2.28–7.46]). The risk of elevated HOMA-IR was significantly higher in TG-N (adjusted OR 2.16 [95% CI 1.33–3.50]) and TG-WC (adjusted OR 2.04 [95% CI 1.22–3.40]). The risk of elevated HbA1c was significantly higher in the TG-WC (adjusted OR 2.79 [95% CI 1.47–5.31]). Hypertriglyceridemic waist phenotype can be a potential and cost-effective method to identify individuals with a high risk of fatty liver and glycometabolic disorders.

## Introduction

The COVID-19 pandemic has re-focused the adverse effects of obesity and diabetes on the general health status of individuals. There are growing concerns that patients with obesity and diabetes are more likely to have severe evolution and mortality during the COVID-19 pandemic^1^. It has also been speculated that there is less effectiveness and more risk following vaccination in preventing 2019 novel coronavirus among obese individuals^2,3^. These remind us that obesity and diabetes have brought pathophysiological changes to the biological systems of the human body. Therefore, there is a high priority to develop specific phenotypes of obesity and diabetes to better assess the risks and efficient screening, and to further engage patients into individualized treatments.

Heterogeneous subgroups among the overweight/obese based on phenotypic differences for metabolic risk factors have been observed previously^4^. The complexity of phenotypes may contribute to some of the variations in symptomatic outcomes of diabetes, fatty liver, and mortalities related to obesity. Obesity, especially increases in visceral fat deposits, have been recognized as one of the leading causes of all metabolic risk factors^5^. However, overweight/obese individuals with similar levels of adiposity measured by body mass index (BMI) or waist circumference (WC) can display different metabolic profiles characterized by lipid, insulin, and glucose concentrations^6^. Insulin resistance has elucidated most of the pathophysiology of the metabolic syndrome with a significant contribution to hyperglycemia^7^. Furthermore, a major contributor to the development of insulin resistance is an overabundance of circulating fatty acids mainly derived from triglyceride^8^. Hence, the main factors to identify high risks of obesity and diabetes are visceral fat and abnormality in triglyceride concentrations.

The hypertriglyceridemic waist phenotype is characterized by the simultaneous presence of elevated serum triglyceride (TG) levels with an increased WC. Hypertriglyceridemic waist phenotype has been proposed as a cost-effective method of screening in clinical practice to overcome the major barriers of the high cost of measuring visceral adiposity and insulin^9^. Previous studies conducted with overweight/obese populations, found a higher risk of diabetes in such phenotype among the elderly^10–12^. It was also suggested that hypertriglyceridemic waist was more prevalent in young and middle-aged adults^10^. Fatty liver is considered to be a hepatic injury associated with the metabolic syndrome in parallel with obesity and diabetes^13^. The prevalence of fatty liver has continued to grow in recent years. Most of the patients with fatty liver disease are asymptomatic and ignored. The diagnosis of fatty liver is complicated and is depended on abnormal imaging results and liver enzyme activity following biopsy examinations, which are also expensive and invasive^14^. The association between the hypertriglyceridemic waist phenotype and fatty liver disease needs further scientific investigation.

The purpose of this study was to distinguish the relationships between the hypertriglyceridemic waist phenotype and the risk of developing fatty liver and associated abnormal glycometabolic profiles in overweight/obese adults. It was hypothesized that the hypertriglyceridemic waist phenotype could be associated with a high prevalence of fatty liver and glycometabolic disorders.

## Methods

### Study population

Participants were recruited by convenient sampling via fliers posted and distributed in local urban communities of the Haidian District, Beijing, China, from April to May 2014. Inclusion criteria were: (1) resided locally for a continuous period of not less than 1 year; (2) 20–55 years; (3) a nutritional status of overweight (with BMI ≥ 24.0 kg/m^2^)^15^; (4) no history of severe diseases in the major organs (e.g. heart, liver, kidney, lung, and locomotor function) and no secondary obesity by self-report; (5) not using medications that might impact the measurements obtained in the current study. These included lipid-lowering drugs, hypoglycemic agents, and antihypertensive medications.

Following initial screening, every participant underwent a physical examination including anthropometric measurement, assessment of biomarkers (lipids and glycometabolic profile) from venous blood samples, and ultrasonography to diagnosis fatty liver. The protocol used has been outlined previously in detail^16^. All examinations were conducted by trained doctors, nurses, and sonographers, and daily calibrations of the instruments prior to use was a prerequisite prior to experimental measurements. Anthropometric and ultrasonography measurements were taken twice to establish mean values for statistical analysis. Lifestyle investigation included smoking habits and alcohol consumption (yes or on in the previous week prior to data collection) by self-report. Physical activity and sedentary time (International Physical Activity Questionnaire-Short Form [IPAQ-SF]) were also recorded for all participants. The study was approved by the Ethical Committee for the Use of Human and Animal Subjects in Research by the local university ethics committee (IRB00001052-130 86). Subjects were fully informed about the purpose and constraints of this study prior to data collection, and all participants provided their written informed consent.

### Anthropometric measurements

Participants were instructed to stand up-right in a vertical position in bare feet, wearing light clothing for all anthropometric measures. Weight was measured with a lever scale (model RGT-140, Bengbu Hengsheng Medical Equipment Co., China) to the nearest 0.1 kg; height with a stadiometer (model TZG, Bengbu Hengsheng Medical Equipment Co., China) to the nearest 0.1 cm. BMI was calculated as weight in kilograms divided by height in meters squared. Overweight was defined as 24.0 ≤ BMI < 28.0kg/m^2^, obesity as BMI ≥ 28.0 kg/m^2^^15^.

WC was measured at the midpoint between the lowest rib and iliac crest with a Myo Tape measuring tape (Accufitness, Green Village, USA) to the nearest 0.1 cm, at the hipline at the widest part of hips. High WC was defined as ≥ 102.0 for males and ≥ 88.0 cm for females^10,17,18^.

BP was measured in a seated position following 5 min of rest with a clinical sphygmomanometer (model XJ1ID, Yutu, China) and a stethoscope (model TZ-1, Yuyue, China) to the nearest 1 mm Hg. High BP was defined as systolic BP (SBP) ≥ 140 and/or diastolic BP (DBP) ≥ 90 mmHg.

### Assessment of biomarkers

All participants were overnight fasted for 8–12 h prior to a venous blood draw in the morning. TG, total cholesterol (TC), high-density lipoprotein cholesterol (HDL), low-density lipoprotein cholesterol (LDL), and glucose were measured using commercial assay kits (Yingkexinchuang Institute, China) by a spectrophotometer (AU400, Olympus, Japan), as well as glycosylated hemoglobin (HbA1c, Kemei Biotechnology Co., China). High TG was defined as ≥ 1.71 mmol/L^10,17,18^, high glucose as ≥ 5.6 mmol/L^19^ and high HbA1c as ≥ 6.5%^20,21^.

Insulin was measured using the radioimmunoassay technique (Model XH-6080, Xian Nuclear Instruments Co., China). The Homeostatic Model Assessment of Insulin Resistance (HOMA-IR) index was calculated as outlined previously^22^. Briefly the calculation was HOMA-IR = blood glucose (mmol/L) × insulin (mU/L)/22.5.

High HOMA-IR was defined as ≥ 3.19^23^.

### Diagnosis of fatty liver

Real-time ultrasonography (Logiq 180, GE, USA) was used to identify fatty liver. Fatty liver was diagnosed according to the criteria of the presence of at least 2 of 3 abnormal findings in the abdominal area: (1) diffusely increased echogenicity liver (bright liver with its echogenicity greater than the kidney); (2) vascular blurring; (3) gradual attenuation of ultrasound echo^24^.

### Definition of phenotypes

Participants were classified into four phenotypes (1) G1: N-N, normal TG & normal WC; (2) G2: N-WC, normal TG & high WC; (3) G3: TG-N, high TG & normal WC; (4) G4: TG-W, high TG & high WC.

### Statistical analysis

Data were analyzed using IBM SPSS for Windows (version 20.0, SPSS Inc., USA). Continuous variables were expressed as mean (standard deviation [SD]) and categorical variables as n (percent%). One-way analysis of variance and chi-square tests were used for the comparison of continuous variables and categorical variables among the four phenotypes. Logistic regression models were used to analyze the association between phenotypes and risk of fatty liver or glycometabolic profiles, while odds ratio (OR) with 95% confidence intervals (95% CI) in different models were calculated. Model 1 was a crude model without adjustment, Model 2 was adjusted for age, sex, and BMI, and Model 3 was further adjusted for smoking, alcohol consumption, physical activity, and sedentary time. Following control of potential confounders, the association was further explored by subgroup analysis, which was stratified based on sex (male or female), BMI (overweight or obesity), and BP (normal or high), using the normal TG and WC group as a reference. Two-tailed *p* < 0.05 was the level set for statistical significance.

### Ethics approval and consent to participate

All procedures performed in the present study were in accordance with the Declaration of Helsinki and were approved by the Medical Ethical Committee of the Peking University Health Science Center (IRB00001052-13086). The author obtained permission to use the dataset from Institute of Child and Adolescent health, Peking University and the secondary analysis of the dataset without identifiable information was approved by the Ethical Committee of Hunan Normal University (NO.2019-88). Before the formal investigation, written informed consent to participate in the study was obtained from all participants. Also, all participants had the right to withdraw from the study at any time and for any reason.

## Results

### General characteristics of the study population

Finally, 1221 participants were involved in the current study. General characteristics were as follows: mean (SD) age of 37 (9) years, n (percent%) of 455 (37.3%) males and 766 (62.7%) females, BMI 29.0 (4.0) kg/m^2^, TG 2.04 (1.45) mmol/L, WC 95.8 (10.7) cm, and the prevalence of fatty liver was 74.3% totally. Table 1 shows the differences in anthropometric measurements, assessment of biomarkers (fasting lipids and glycometabolic profiles), diagnosis of fatty liver, and lifestyle among the four phenotypes. Participants in G4 group develped the highest BMI, WC, BP, LDL, insulin, HOMA-IR, HbA1c, and fatty liver.

**Table 1:** General characteristics of participants with different phenotypes.

Variables	Category	G1 N-N n = 295	G2 N-WC n = 327	G3 TG-N n = 253	G4 TG-WC n = 346	*P* value
Age (y)		35 (9)	37 (9)	37 (9)	37 (9)	0.07
Sex	Male	106 (35.9%)	47 (14.4%)	171 (67.6%)	131 (37.9%)	< 0.001
	Female	189 (64.1%)	280 (85.6%)	82 (32.4%)	215 (62.1%)	
**Anthropometry**						
BMI (kg/m^2^)		26.6 (1.8)	30.7 (4.1)	27.5 (2.1)	31.8 (4.1)	< 0.001^a,b,c,d,e,f^
WC (cm)		87.1 (6.0)	99.2 (9.6)	91.0 (6.6)	103.4 (10.3)	< 0.001^a,b,c,d,e,f^
Hipline (cm)		98.9 (4.3)	106.1 (7.8)	98.7 (4.7)	106.6 (9.6)	< 0.001^a,c,d,f^
SBP (mmHg)		120 (13)	124 (15)	125 (15)	128 (16)	< 0.001^a,b,c,e,f^
DBP (mmHg)		80 (9)	84 (11)	85 (11)	87 (12)	< 0.001^a,b,c,e,f^
**Fasting lipids profile**						
TG (mmol/L)		1.22 (0.28)	1.28 (0.27)	2.86 (1.37)	2.84 (1.92)	< 0.001^b,c,d,e^
TC (mmol/L)		4.66 (0.83)	4.68 (0.90)	5.26 (0.96)	5.32 (0.89)	< 0.001^b,c,d,e^
HDL (mmol/L)		1.28 (0.25)	1.23 (0.25)	1.18 (0.22)	1.16 (0.20)	< 0.001^a,b,c,d,e^
LDL (mmol/L)		2.57 (0.66)	2.68 (0.73)	2.89 (0.73)	3.02 (0.76)	< 0.001^b,c,d,e,f^
**Glycometabolic profile**						
Insulin (uIU/ml)		9.67 (8.67)	12.67 (9.40)	12.69 (10.93)	17.18 (13.10)	< 0.001^a,b,c,e,f^
HOMA-IR		2.26 (2.10)	3.13 (2.93)	3.19 (3.67)	4.45 (3.99)	< 0.001^a,b,c,e,f^
Glucose (mmol/L)		5.2 (0.6)	5.4 (1.2)	5.5 (1.1)	5.7 (1.5)	< 0.001^b,c,e^
HbA1c (%)		5.6 (0.3)	5.7 (0.6)	5.7 (0.7)	5.9 (0.8)	< 0.001^a,b,c,e,f^
High HOMA-IR	No	244 (82.7%)	218 (66.7%)	167 (66.0%)	169 (48.8%)	< 0.001^a,b,c,e,f^
	Yes	51 (17.3%)	109 (33.3%)	86 (34.0%)	177 (51.2%)	
High glucose	No	178 (60.3%)	131 (40.1%)	171 (67.6%)	156 (45.1%)	< 0.001^a,b,c,d,f^
	Yes	117 (39.7%)	196 (59.9%)	82 (32.4%)	190 (54.9%)	
High HbA1c	No	273 (92.5%)	283 (86.5%)	214 (84.6%)	236 (68.2%)	< 0.001^a,b,c,e,f^
	Yes	22 (7.5%)	44 (13.5%)	39 (15.4%)	110 (31.8%)	
Fatty liver	No	159 (54.8%)	53 (16.4%)	56 (22.6%)	31 (9.0%)	< 0.001^a,b,c,e,f^
	Yes	131 (45.2%)	271 (83.6%)	192 (77.4%)	314 (91.0%)	
**Lifestyle investigation**						
Smoke	No	171 (79.5%)	203 (88.6%)	128 (63.1%)	192 (74.4%)	< 0.001^a,b,d,e,f^
	Yes	44 (20.5%)	26 (11.4%)	75 (36.9%)	66 (25.6%)	
Alcohol	No	168 (78.1%)	193 (84.3%)	136 (67.0%)	198 (76.7%)	< 0.001^d^
	Yes	47 (21.9%)	36 (15.7%)	67 (33.0%)	60 (23.3%)	
PA (MET-min/week)^g^		1655 (1385)	1866 (1570)	1756 (1575)	1432 (1396)	0.01^e,f^
Sedentary time (min/day)^g^		360 (193)	345 (194)	370 (194)	378 (194)	0.28

Data expressed as mean (standard deviation [SD]) or n (percent%).

G1, N-N, normal TG & normal WC; G2, N-WC, normal TG & high WC; G3, TG-N, high TG & normal WC; G4, TG-WC, high TG & high WC.

BMI, body mass index; WC, waist circumference; SBP, systolic blood pressure; DBP, diastolic blood pressure; TG, triglyceride; HOMA-IR, Homeostatic Model Assessment of Insulin Resistance index; HbA1c, glycosylated hemoglobin; TC, total cholesterol; HDL, high density lipoprotein cholesterol; LDL, low density lipoprotein cholesterol; PA, physical activity.

^a,b,c,d,e,f^*P* < 0.05 for G2 vs G1, G3 vs G1, G4 vs G1, G3 vs G2, G4 vs G2, and G4 vs G3, respectively; g, valid n of participants for G1-4 groups were 215, 229, 203, and 258, respectively.

High WC was defined as ≥ 102.0 cm for males and ≥ 88.0 cm for females, high TG as ≥ 1.71 mmol/L, high HOMA-IR as ≥ 3.19, high glucose as ≥ 5.6 mmol/L, and high HbA1c as ≥ 6.5%.

Overweight was defined as 24.0 ≤ BMI < 28.0 kg/m^2^, obesity as BMI ≥ 28.0 kg/m^2^.

### Risk of fatty liver and glycometabolic disorder by phenotypes and associated subgroup analysis

As shown in Table 2, risks of fatty liver were detected in G2-4, high HOMA-IR in G3-4, high HbA1c in the G4. These associations were consistently observed with or without additional adjustment for potential confounders.

**Table 2:** Association of hypertriglyceridemic waist phenotype and fatty liver and glycometabolic profile.

Variables	Case/n	Model 1	Model 2	Model 3
**Fatty liver**				
G1, N-N	131/290	1 (reference)	1 (reference)	1 (reference)
G2, N-WC	271/324	6.21 (4.27–9.02)	2.80 (1.77–4.43)	3.50 (2.05–5.97)
G3, TG-N	192/248	4.16 (2.86–6.07)	2.50 (1.64–3.81)	2.59 (1.61–4.16)
G4, TG-WC	314/345	12.29 (7.96–19.00)	3.38 (2.01–5.68)	4.12 (2.28–7.46)
**High HOMA-IR**				
G1, N-N	51/295	1 (reference)	1 (reference)	1 (reference)
G2, N-WC	109/327	2.39 (1.64–3.50)	1.36 (0.88–2.12)	1.34 (0.80–2.26)
G3, TG-N	86/253	2.46 (1.65–3.67)	2.14 (1.41–3.25)	2.16 (1.33–3.50)
G4, TG-WC	177/346	5.01 (3.47–7.24)	2.50 (1.62–3.83)	2.04 (1.22–3.40)
**High glucose**				
G1, N-N	117/295	1 (reference)	1 (reference)	1 (reference)
G2, N-WC	196/327	1.46 (1.01–2.11)	0.90 (0.58–1.37)	1.00 (0.61–1.63)
G3, TG-N	82/253	1.86 (1.27–2.73)	1.63 (1.09–2.43)	1.53 (0.97–2.41)
G4, TG-WC	190/346	2.16 (1.52–3.08)	1.23 (0.81–1.87)	1.20 (0.74–1.95)
**High HbA1c**				
G1, N-N	22/295	1 (reference)	1 (reference)	1 (reference)
G2, N-WC	44/327	1.93 (1.13–3.30)	1.10 (0.60–2.02)	1.13 (0.57–2.25)
G3, TG-N	39/253	2.26 (1.30–3.93)	1.79 (1.01–3.17)	1.77 (0.94–3.34)
G4, TG-WC	110/346	5.78 (3.54–9.44)	3.11 (1.78–5.43)	2.79 (1.47–5.31)

Data expressed as odds ratio (OR) ([95% confidence interval [95% CI]).

Model 1 is crude model without adjustment; Model 2 adjusted for age, sex and body mass index (BMI); Model 3 further adjusted for potential lifestyle (smoke, alcohol, physical activity and sedentary time);

G1, N-N, normal TG & normal WC; G2, N-WC, normal TG & high WC; G3, TG-N, high TG & normal WC; G4, TG-WC, high TG & high WC.

HOMA-IR, Homeostatic Model Assessment of Insulin Resistance index; HbA1c, glycosylated hemoglobin.

High WC was defined as ≥ 102.0 cm for males and ≥ 88.0 cm for females, high TG as ≥ 1.71 mmol/L, high HOMA-IR as ≥ 3.19, high glucose as ≥ 5.6 mmol/L, and high HbA1c as ≥ 6.5%.

Overweight was defined as 24.0 ≤ BMI < 28.0 kg/m^2^, obesity as BMI ≥ 28.0 kg/m^2^.

**Figure 1:**
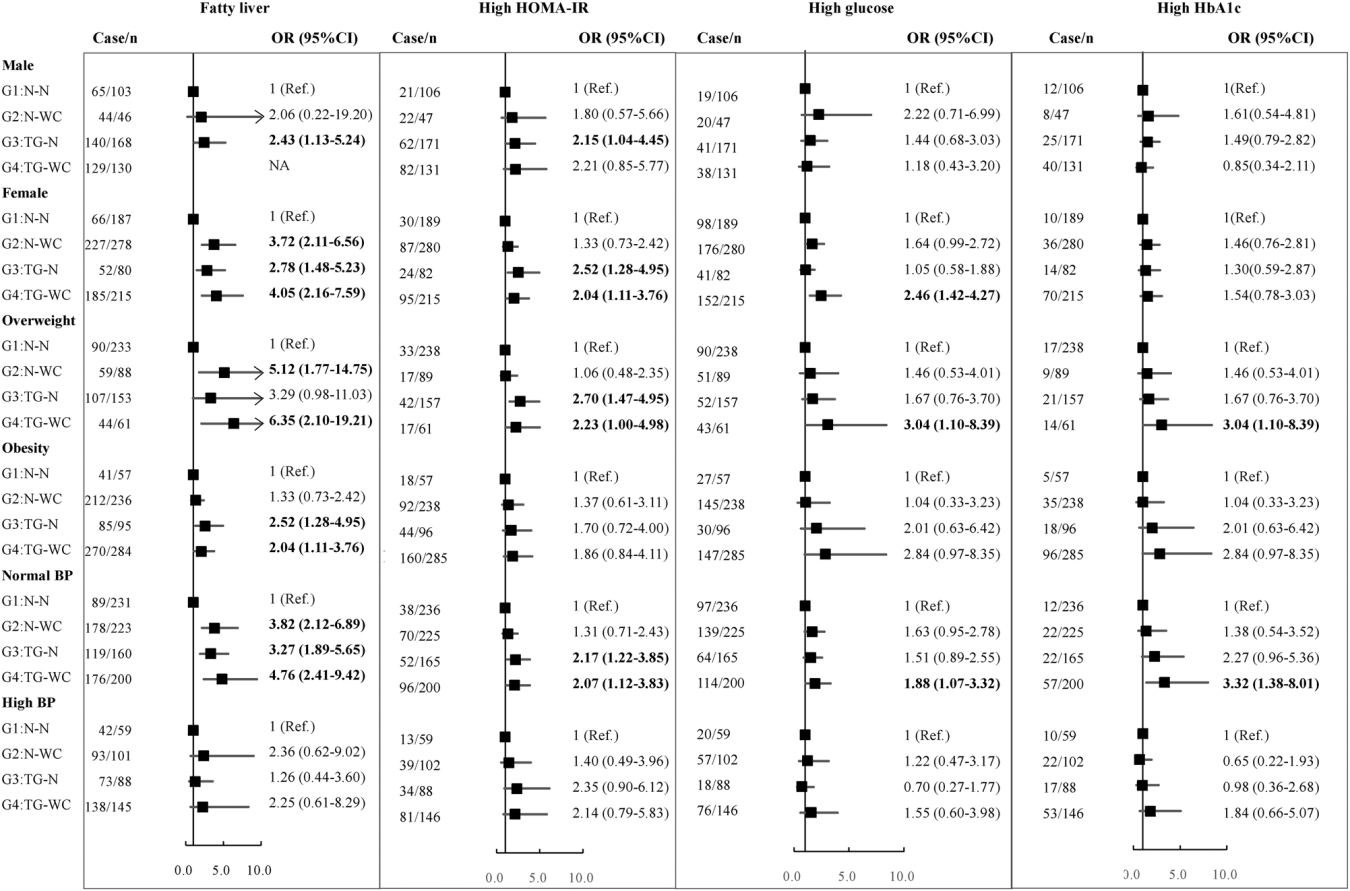
Subgroup analysis of the association between hypertriglyceridemic waist phenotype and fatty liver and glycometabolic profile. Data expressed as odds ratio (OR) ([95% confidence interval [CI]); All the analyses were adjusted for age, sex, body mass index (BMI), smoke, alcohol, physical activity, and sedentary time; G1, N-N, normal TG & normal WC; G2: N-WC, normal TG & high WC; G3: TG-N, high TG & normal WC; G4: TG-WC, high TG & high WC; HOMA-IR, Homeostatic Model Assessment of Insulin Resistance index; HbA1c, glycosylated hemoglobin; BP, blood pressure; High WC was defined as ≥ 102.0 cm for males and ≥ 88.0 cm for females, high TG as ≥ 1.71 mmol/L, high HOMA-IR as ≥ 3.19, high glucose as ≥ 5.6 mmol/L, and high HbA1c as ≥ 6.5%; Overweight was defined as 24.0 ≤ BMI < 28.0 kg/m^2^, obesity as BMI ≥ 28.0 kg/m^2^.

As shown in Fig. 1, associations between phenotypes and the risk of fatty liver and glycometabolic disorder including high HOMA-IR, glucose and HbA1c were examined by subgroups: sex (male or female), BMI (overweight or obesity), BP (normal or high), with G1 used as a reference. The identification ability of hypertriglyceridemic waist phenotype was more pronounced in females, and also in the overweight and normal BP subgroups.

## Discussion

In the current cross-sectional study, we found that the hypertriglyceridemic waist phenotype was independently associated with a 4.1-fold higher risk to develop fatty liver, twofold increased insulin resistance, and 2.8-fold elevated HbA1c than those with normal TG and WC in Chinese overweight/obese adults. We further observed that the relevant risks associated with the hypertriglyceridemic waist phenotype could be 1.9- to 6.4-fold higher especially in females, the overweight, and non-hypertensive subgroups. The phenotype for high TG but normal WC, was associated with 2.6- and 2.2-fold high prevalence of fatty liver and insulin resistance. The strength and novelty of this study was the finding that the hypertriglyceridemic waist phenotype was a cost-effective screening tool for fatty liver and diabetes identification among overweight/obese adults.

The concept of the hypertriglyceridemic waist phenotype was first proposed by Lemieux et al. based on the evidence that high TG or WC alone were inadequate to identify the metabolic triad^9^. This phenotype has been widely accepted in risk assessment or prediction of high visceral fat^25^, diabetes^26^, hypertension^27^, metabolic syndrome^28^, atherosclerosis^29^, cardiovascular diseases^30^, and renal dysfunction^31^. All these results support that the hypertriglyceridemic waist phenotype is a cost-effective screening tool that can be used in clinical practice and is an efficient and effective phenotype that can be used for health management. However, the application of this phenotype in the identification of fatty liver disease is still limited. Previously, a cross-sectional study focusing on children/adolescents found that hypertriglyceridemic waist phenotype was related to non-alcoholic fatty liver disease^32^. In the adult population, Liu et al. demonstrated that phenotypes combining TG and BMI were a better indicator of fatty liver disease among premenopausal women, and that WC alone was associated with the disease in postmenopausal women^33^. This finding was also supported by Yu et al. who observed that the hypertriglyceridemic waist phenotype was significantly associated with a higher risk of abnormal liver function in Chinese adults^34^. In our study population, overweight/obese adults suffered from visceral adiposity and glycometabolic disorders, that are conditions closely linked to fatty liver disease^35^. Interestingly, during the subgroup analysis, the identification ability of hypertriglyceridemic waist phenotype for fatty liver and abnormal glycometabolic profile was less effective in the obese and hypertension subgroups. We speculate that lower heterogeneities and small sample size may have contributed to the decrease in predictive validities.

We also found an association between hypertriglyceridemic waist phenotype and insulin resistance, which was in agreement with some of the previous studies that investigated abnormal glucose metabolism in female, overweight, and normal subgroups. A meta-analysis concluded that participants with the hypertriglyceridemic waist phenotype had more serious insulin resistance problems and higher glucose concentrations^36^. In our study, following adjustment for potential confounders, the risk of high glucose was not significantly different for phenotypes. This may be the result of the regulation of β cells. At the early stage of insulin resistance, β cells can increase secretion to maintain normal glucose tolerance^37^. Elevated blood glucose can appear when β cells cannot release sufficient insulin. Further to this, some of the studies concentrated on the hypertriglyceridemic waist phenotype and levels of HbA1c, but there were no studies that demonstrated a specific association between phenotypes^38,39^. The findings of this study demonstrate that the hypertriglyceridemic waist phenotype was associated with a high level of HbA1c concentration especially in the overweight and normal BP populations.

There are several limitations in the current study that need consideration. A relatively small sample size and wide confidence intervals around the estimates of associations may have resulted in low predictive ability within the subgroups of the males, the obese, and the hypertensive. It can also be speculated that there was relatively low sensitivity and specificity of the hypertriglyceridemic waist phenotype in the Chinese population sampled due to the relatively low TG and WC observed^34^. Referring to the diagnosis of fatty liver, imaging results and liver enzyme activity following biopsy examinations, which are gold standard, were not used in the current study. In addition, we were not able to categorize non-alcoholic fatty liver disease which might lead to a bias in the findings. However, we included alcohol as a confounder, and the associations were persistently significant. The target population in our study was Chinese overweight/obese adults, which may not represent normal weight adults and different populations. The clinical relevance of our findings, while exciting, need further experimental investigation using larger population samples and sub group analysis.

## Conclusion

The hypertriglyceridemic waist phenotype was associated with risks of developing fatty liver and glycometabolic disorders in overweight/obese Chinese adults especially in female, overweight, and non-hypertensive subgroups.

## Data Availability

The datasets collected and analyzed in our study are available from the corresponding author (Yide Yang, email: yangyide@hunnu.edu.cn) on reasonable request.
